# Expression of G*α*q Is Decreased in Lymphocytes from Primary Sjögren's Syndrome Patients and Related to Increased IL-17A Expression

**DOI:** 10.1155/2018/8212641

**Published:** 2018-06-07

**Authors:** Yuechi Sun, Ying Wang, Shiju Chen, Guihua Fan, Junhui Zhang, Fan Dai, Hongyan Qian, Yuan Liu, Guixiu Shi

**Affiliations:** ^1^Department of Rheumatology and Clinical Immunology, The First Affiliated Hospital of Xiamen University, Xiamen 361003, China; ^2^The Chenggong Hospital Affiliated to Xiamen University, Xiamen 361003, China; ^3^Xiamen Key Laboratory of Rheumatology and Clinical Immunology, Xiamen 361003, China

## Abstract

Primary Sjögren's syndrome (pSS) is a rheumatic disease characterized by the destruction of salivary and lacrimal glands, and its pathogenesis mechanism remains unclear. G*α*q is the *α*-subunit of the heterotrimeric Gq protein. Researches demonstrated that G*α*q was involved in the pathogenesis regulation of several rheumatic diseases. This study explored the role of G*α*q in pSS. G*α*q mRNA levels in peripheral blood mononuclear cells (PBMCs) from 39 patients and 26 healthy controls (HCs) were investigated using real-time PCR. IL-17A serum concentrations in 22 pSS patients and 23 HCs were tested by ELISA, and the clinical significance of G*α*q was analyzed. The association of G*α*q with interleukin-17A (IL-17A) expression was also analyzed in patients with pSS. We showed that G*α*q expression in PBMCs from patients with pSS was significantly lower than that in PBMCs from HCs. G*α*q expression level was closely associated with pSS disease activity. Furthermore, a negative association was also found in IL-17A and G*α*q expression level. These data suggest that G*α*q is involved in pSS pathogenesis regulation, possibly due to its regulation of Th17. These results provide new insights into the pSS pathogenesis mechanism involving abnormal Th17 regulation.

## 1. Introduction

Primary Sjögren's syndrome (pSS), one of the most common rheumatic diseases, is characterized by dry eyes and dry mouth, due to lymphoplasmocytic infiltration and destruction of lachrymal and salivary glands. In addition to the hypofunction of the salivary and lacrimal glands, other vital organs such as the lungs, kidneys, and liver can also be damaged in patients with pSS [1].

Specific details regarding the etiology of pSS remain unknown. The diagnosis of pSS is always at a relatively late stage when irreversible damages of the glands already existed, and the effective treatment strategies are limited compared with other rheumatic diseases. Intensive studies are trying to unravel the molecular, genetic, and immunological mechanisms for this disease and provide a better understanding about the pathogenesis mechanism of pSS [2]. T helper type 17 (Th17), characterized by the production of IL-17, is a subset of effector T helper cells that are distinct from Th1 and Th2 cells [3]. They have been proven to be the main pathogenic cells in inflammation and autoimmunity [4]. Several studies have indicated that Th17 cells are increased in patients with pSS and involved in the glandular tissue damage of SS [5–7]. However, the presence of Th17 is always found to be associated with the onset of gland destruction; how Th17 is regulated in pSS remains unclear. A study about the mechanism of Th17 regulation in pSS may help us have a better understanding of pSS at an early stage before the onset of gland destruction and may help us explore more treatment targets in pSS.

G*α*q is encoded by gene GNAQ; it is the *α*-subunit of the heterotrimeric Gq protein. The Gq protein is a member of the subfamilies of the heterotrimeric G proteins. There are three subunits in the heterotrimeric G proteins, namely, *α*, *β*, and *γ*. According to the difference of the *α*-subunits, the G proteins can be classified into four subfamilies including G*α*s, G*α*i, G*α*q/11, and G12/13. Gq is a member of the G*α*q/11 subfamily [8]. G*α*q is widely expressed in several kinds of cells, including lymphocytes. G*α*q couples with a wide variety of membrane receptors to effector molecules inside cells. Important roles of G*α*q in the immune system have been revealed in recent years, giving us new understanding about the pathogenesis mechanism of rheumatic diseases [9, 10]. Our previous researches demonstrated that G*α*q is involved in the pathogenesis regulation of several rheumatic diseases, including systemic lupus erythematosus (SLE) and rheumatoid arthritis (RA), which may be related to its regulation in Th17 differentiation [11, 12]. pSS shares some similarities with the pathogenesis mechanisms of SLE or RA; however, whether G*α*q is also involved in pSS pathogenesis remains unknown.

In this study, we investigated the expression of G*α*q in patients with pSS and analyzed the association of G*α*q expression and the clinical characteristics of patients with pSS. We then studied the level of IL-17A in the serum of patients with pSS and the association between IL-17A and G*α*q expression. We found the expression of G*α*q was significantly lower in patients with pSS and that the expression of G*α*q was associated with the disease activity of pSS, presence of arthritis, and IgG level. The concentration of IL-17A was significantly higher in patients with pSS, and the expression of G*α*q was negatively related to IL-17A. Our data suggest that G*α*q is involved in pSS pathogenesis regulation, indicating that G*α*q can be used as a potential target in pSS research and treatment.

## 2. Materials and Methods

### 2.1. Patients and Controls

39 patients diagnosed with pSS (22 pSS patients with both PBMCs and sera) and 40 healthy controls (HCs, 17 HCs with samples of PBMCs, 14 HCs with samples of sera, and 9 HCs with samples of both PBMCs and sera) matched for sex and age were included in this study. Patients with pSS were all from the outpatient and inpatient departments of Rheumatology and Clinical Immunology, the First Affiliated Hospital of Xiamen University. The patients with pSS were diagnosed based on the criteria of the American College of Rheumatology [13]. Data such as sex, age, history, clinical manifestations, laboratory findings, and treatment strategy on patients with pSS were collected from the patients' medical records. This study was approved by the medical ethics committee of the First Affiliated Hospital of Xiamen University. The basic clinical characteristics of the pSS patients and HCs are summarized in Table 1.

### 2.2. Peripheral Blood Mononuclear Cell Isolation

Peripheral blood mononuclear cells (PBMCs) were isolated from blood samples of HCs and pSS patients using standard density gradient centrifugation. The heparinized blood samples were centrifuged with the addition of Ficoll-Paque Plus (Eppendorf, GER). Total RNA of PBMCs was isolated using TRIzol™ Reagent (Invitrogen, USA). RNA was then reverse transcribed following the manufacturer's instructions. The concentration of RNA was determined with a Nanodrop ND1000 spectrophotometer.

### 2.3. RNA Extraction and Quantification of Transcripts Using Real-Time Polymerase Chain Reaction

Total RNA of PBMCs of patients and HCs was extracted by TRIzol (Invitrogen, USA) according to the manufacturer's instructions. Briefly, total RNA was reverse-transcribed first to cDNA using reverse transcription reagent kits (Roche, CH) following the manufacturer's instructions. The reaction conditions were as follows: 50°C for 60 mins, then 85°C for 5 min, and finally 4°C for 5 min. The mRNA expression levels of GAPDH and G*α*q were investigated by real-time quantitative PCR (RT-PCR) using SYBR Green (Roche, CH). A 10 *μ*L SYBR Green RT-PCR reaction mixture containing 2 *μ*L of cDNA, 0.2 *μ*L of sense or antisense primer, and 7.6 *μ*L ddH2O was used. Quantitative PCR was performed according to the manufacturer's instructions (ABI7500, USA). The PCR reactions were amplified as follows: 50°C for 2 min, 95°C for 10 min, 40 cycles at 95°C for 15 s and 60°C for 1 min, and 95°C for 1 min to denature. GAPDH expression level was used to normalize the mRNA expression level of G*α*q; the 2^−ΔΔCt^ method was used to determine the relative expression. The primers used were as follows: GAPDH, sense primer: 5′-GTGAACCATGAGAAGTATGACAAC-3′ and antisense primer: 5′-CATGAGTCCTTCCACGATACC-3′ and G*α*q, sense primer: 5′- GTTGATGTGGAGAAGGTGTCTG-3′ and anti-sense primer: 5′-GTAGGCAGGTAGGCAGGGT-3′. To ensure the amplification specificity, melting curve analysis was used in the PCR products from each primer pair and then agarose gel electrophoresis was used.

### 2.4. Enzyme-Linked Immunosorbent Assay (ELISA)

The concentration of L-17A in the serum of HCs and pSS patients was determined by an ELISA kit (PeproTech, USA) following the manufacturer's protocols. An ELISA microplate reader was used to measure the absorbance at 450 nm.

### 2.5. Statistical Analysis

The results were analyzed using the Mann–Whitney *U* test with PRISM software (GraphPad Software, San Diego, CA, USA). Spearmen's rank test was used to investigate the correlations between the clinical parameters of patients with pSS and G*α*q expression level. *P* values < 0.05 were considered significant.

## 3. Results

### 3.1. Expression of G*α*q Was Decreased in Patients with pSS

**Figure 1:**
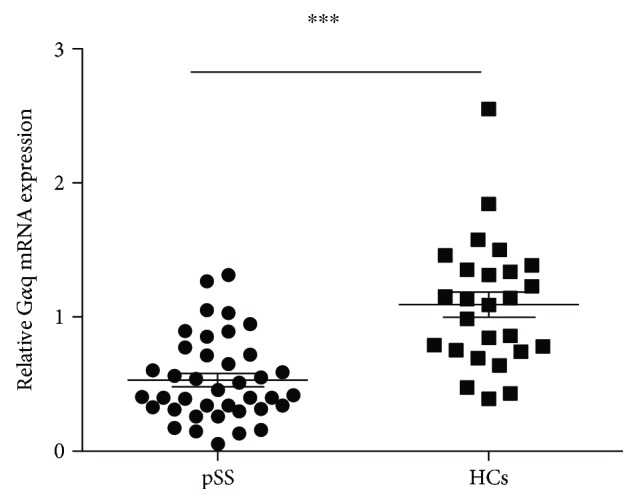
Expression of G*α*q was decreased in lymphocytes of pSS patients. Peripheral blood mononuclear cells (PBMCs) from pSS (*n* = 39) and HC (*n* = 26) were collected. G*α*q mRNA expression in PBMCs was analyzed by RT-PCR. The mRNA expression level of G*α*q was significantly higher in pSS patients compared with HCs. ^∗∗∗^*P* < 0.001.

To explore the role of G*α*q in pSS, we first investigated the G*α*q expression level in patients with pSS. As an imbalance in lymphocytes is a main factor in pSS pathogenesis, we collected PBMCs from patients with SS and HCs and analyzed the mRNA expression of G*α*q in PBMCs. As shown in Figure 1, we found that the mRNA expression of G*α*q in PBMCs was significantly lower in patients with SS compared with that in HCs. These data suggest G*α*q might have a potential role in the regulation of SS pathogenesis (Figure 1).

### 3.2. Expression of G*α*q Was Negatively Associated with Disease Activity in Patients with pSS

**Figure 2:**
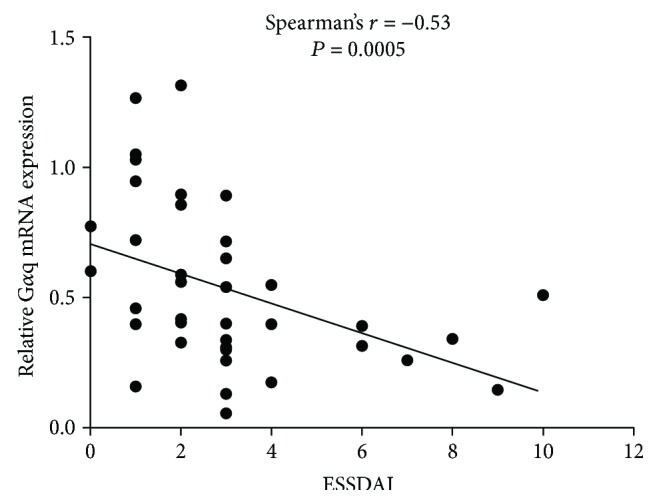
Expression of G*α*q was negatively associated with the disease activity of pSS patients. Peripheral blood mononuclear cells (PBMCs) from pSS (*n* = 39) patients were collected. G*α*q mRNA expression in PBMCs was analyzed by RT-PCR. The correlation between G*α*q mRNA expression level and ESSDAI in pSS patients was determined by using Spearmen's rank test.

To further investigate the specific role of G*α*q in pSS pathogenesis regulation, we then analyzed the disease activity of patients with pSS with different expression levels of G*α*q. The EULAR Sjögren's syndrome disease activity index (ESSDAI) is now widely used to evaluate pSS disease activity [14]. We analyzed the relation of G*α*q mRNA expression in PBMCs with the ESSDAI in patients with pSS. As shown in Figure 2, we found the expression level of G*α*q mRNA was negatively related to the ESSDAI in patients with pSS. This suggested that G*α*q negatively regulates the pathogenesis of pSS and that a low level of G*α*q might contribute to the disease onset of pSS.

### 3.3. Clinical Significance of G*α*q in Patients with pSS

**Figure 3:**
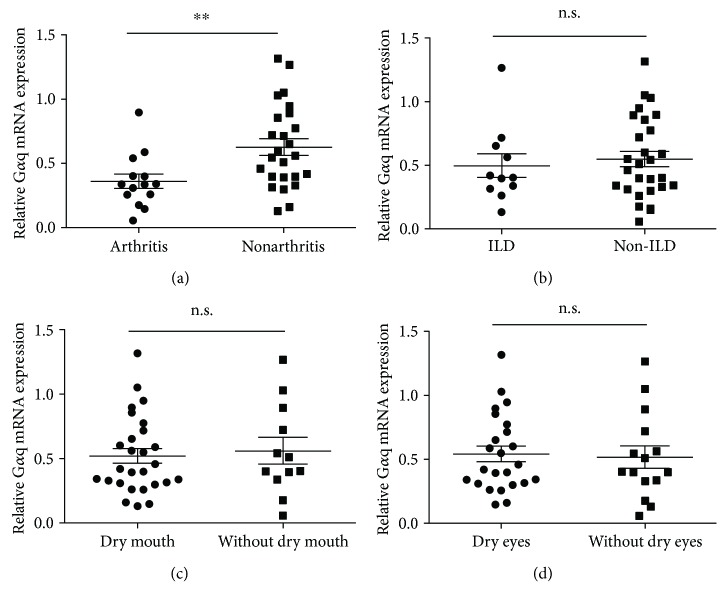
Expression level of G*α*q was much more lower in pSS patients with arthritis. G*α*q mRNA expression in PBMCs of pSS (*n* = 39) patients was analyzed by RT-PCR. (a) G*α*q mRNA expression in pSS patients with and without arthritis. (b) G*α*q mRNA expression in pSS patients with and without interstitial lung diseases (ILD). (c) G*α*q mRNA expression in pSS patients with and without dry mouth. (d) G*α*q mRNA expression in pSS patients with and without dry eyes. ^∗∗^*P* < 0.01.

**Figure 4:**
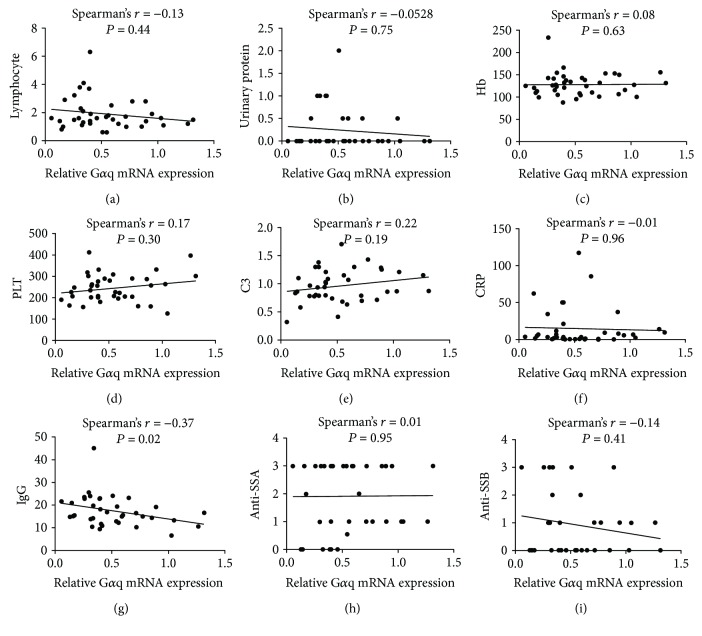
Expression level of G*α*q was negatively associated with IgG level. Association of G*α*q mRNA expression with lymphocyte count in peripheral blood (a), urinary protein (b), level of hemoglobin (HB) (c), platelet (PLT) (d), complement 3 (C3) (e), C-reactive protein (CRP) (f), immunoglobulin G (IgG), titer of anti-SSA (h), and titer of anti-SSB (i). Significant negative association was found between G*α*q mRNA expression and IgG level.

We then analyzed the association of the expression of G*α*q with the clinical characteristics of patients with pSS to explore the clinical significance of G*α*q in pSS. The expression of G*α*q was significantly lower in pSS patients with arthritis than that in those without arthritis (Figure 3(a)), suggesting that G*α*q might be involved in the pathogenesis of arthritis. However, no differences in G*α*q expression between pSS patients with and without interstitial lung disease (ILD), dry eyes, and dry mouth were found (Figures 3(b)–3(d)). We further analyzed the association of G*α*q mRNA expression with other clinical characteristics, including lymphocyte count in peripheral blood, urinary protein, hemoglobin (HB) level, platelet (PLT) level, complement 3 (C3) level, C-reactive protein (CRP) level, immunoglobulin G (IgG) level, anti-SSA titer, and anti-SSB titer (Figure 4). A significant negative association was found between G*α*q mRNA expression and IgG level, whereas no association was found for the others. These data suggest that G*α*q plays a role in the negative regulation of the immune reaction.

### 3.4. G*α*q Expression Was Negatively Related to IL-17A Concentration, Which May Contribute to the Mechanism by Which G*α*q Is Involved in pSS Pathogenesis Regulation

**Figure 5:**
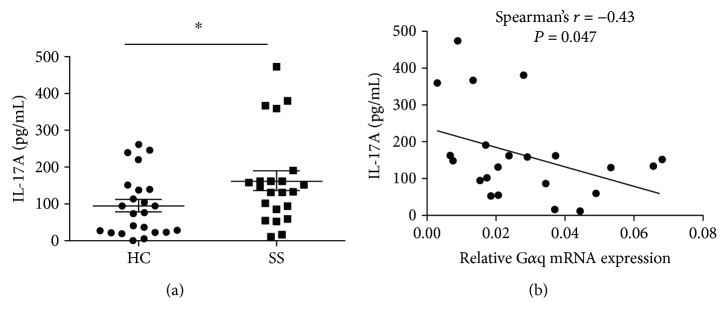
Level of IL-17A was increased in pSS patients and negatively correlated with G*α*q mRNA expression. (a) Level of IL-17A in serum of pSS (*n* = 22) patients and HCs (*n* = 23). Level IL-17A was significantly higher in pSS patients than in HCs. (b) The correlation between G*α*q mRNA expression level and IL-17A in pSS patients (*n* = 22) was determined by using Spearmen's rank test. ^∗^*P* < 0.05.

Our data suggest that G*α*q is involved in pSS pathogenesis regulation, but the mechanism remains unclear. Th17 is a key factor in pSS pathogenesis, and our previous researches demonstrated that G*α*q inhibited the differentiation of Th17 [11]. We further investigated the correlation between G*α*q and Th17 in patients with pSS. We first investigated the level of IL-17A in the serum of patients with pSS and HCs. We found that the concentration of IL-17A was significantly higher in patients with pSS than in HCs (Figure 5(a)). We then analyzed the correlation of G*α*q mRNA expression in PBMCs from patients with pSS with IL-17A. A negative correlation was found between G*α*q mRNA expression and IL-17A expression (Figure 5(b)). These data suggested that G*α*q might be a factor involved in Th17 regulation in patients with pSS.

## 4. Discussion

In this study, we report for the first time that G*α*q mRNA expression in lymphocytes was decreased in patients with pSS and that the expression of G*α*q was closely related to disease activity. G*α*q mRNA expression was negatively associated with IL-17A levels in the serum of patients with pSS. Our study revealed that G*α*q plays a role in pSS pathogenesis regulation, providing a new mechanism for how Th17 cells are regulated in pSS.

pSS is a disorder in which dry eyes and dry mouth occur as a manifestation of immune dysregulation [15, 16]. Dry eyes and mouth are often the first symptoms of SS and may indicate the involvement of other organs, including the salivary glands, lungs, and kidneys. Roughly 5–10% of patients with SS develop lymphoma [17]. Treatment strategies for pSS are relatively limited because the irreversible destruction of the salivary glands is always present when pSS is diagnosed. Studies on the pathogenesis mechanism of pSS are needed to clarify the early stages of pSS. Th17 cells have been shown to be a factor in pSS pathogenesis. IL-17 KO mice were shown to be completely resistant to SS induction, and the adoptive transfer of Th17 cells induced the presence of SS symptoms in immunized IL-17 KO mice rapidly, proving the crucial role of Th17 in pSS pathogenesis [18]. Increased levels of IL-17 were also found in the serum as well as the salivary glands of patients with pSS [6, 19]. Consistent with previous studies, we also found that IL-17A was increased in the serum of patients with pSS, confirming the crucial role of Th17 cells in pSS pathogenesis. However, how the abnormal Th17 cells are regulated in pSS remains unclear. Studies on the regulation mechanism of Th17 cells in pSS can help to better understand the stage before the onset of Th17 cell upregulation and the destruction of salivary glands and help to develop better management strategies for pSS in its early stages.

G*α*q is the *α*-subunit of the Gq protein encoded by gene GNAQ [8]. Our previous studies confirmed the vital role of G*α*q in several aspects of immune regulation such as dendritic cell trafficking, B cell selection, and T cell activation [9, 10, 20]. By using Gnaq−/− chimeric mice by reconstituting lethally irradiated C57BL/6J recipient mice with Gnaq−/− bone marrow, we also revealed the role of G*α*q in the pathogenesis of autoimmune disease. Autoimmunity with multiorgan involvement and arthritis can spontaneously develop in Gnaq−/− chimeric mice [10]. In previous studies, the expression of G*α*q was shown to decrease in lymphocytes from patients with RA and SLE and closely related to disease activity, indicating the role of G*α*q in RA and SLE pathogenesis regulation [11, 12, 21, 22]. Thus, these studies revealed a new mechanism for autoimmune disease pathogenesis regulation.

This study demonstrated the role of G*α*q in pSS. We found that the expression of G*α*q was also decreased in lymphocytes from patients with pSS and that the expression level of G*α*q was closely associated with the disease activity of pSS, presence of arthritis, and high level of IgG. Our previous studies demonstrated that Gnaq−/− chimeric mice spontaneously developed inflammatory arthritis [10], and the expression of G*α*q was shown to be decreased in lymphocytes from RA patients, suggesting that G*α*q is involved in the regulation of inflammatory arthritis development. pSS shares some similarities with RA regarding the pathogenesis mechanism and is often coexisted with RA [23]. Our studies have shown that G*α*q is decreased in both patients with pSS and patients with RA, suggesting that G*α*q might contribute to the overlap in the pathogenesis mechanisms of pSS and RA. Furthermore, we found that G*α*q expression was lower in patients with pSS with arthritis compared with that in those without arthritis, suggesting that a low expression level of G*α*q may be used as a predictor for the presence of arthritis in pSS. However, prospective studies are needed to confirm the ability of the G*α*q level to predict arthritis in pSS.

It was demonstrated that G*α*q could negatively regulate Th17 differentiation in our previous studies, and a negative association was found between the expression of G*α*q and IL-17A in patients with RA [11]. In this study, we also found a negative association between G*α*q and IL-17A, revealing a novel regulation mechanism for the abnormal Th17 levels in patients with pSS. This represents a potential new research target for the early stages of pSS.

In conclusion, this study showed that G*α*q expression is involved in pSS pathogenesis regulation, possibly because of its regulation of Th17 cells. These results provide a new mechanism for pSS pathogenesis regarding abnormal Th17 cell regulation.

## Figures and Tables

**Table 1:** Demographic and clinical characteristics of pSS patients.

	pSS	Health controls	*P*
Number, *N*	39	40	NS
Age (years), mean ± SD	46.1 ± 8	48 ± 9	NS
Sex, M/F	3/36	4/36	NS
Disease duration (years), mean ± SD	2.7 ± 1.2		
Clinical manifestations, *N* (%)			
Oral dry	27 (69)	0 (0)	
Eye dry	24 (62)	0 (0)	
Arthritis	14 (36)	0 (0)	
Pulmonary domain	12 (31)	0 (0)	
Autoimmune hemolytic anemia	13 (43)	0 (0)	
Lymphopenia	5 (13)	0 (0)	
Renal involvement	12 (31)	0 (0)	
Nervous system involvement	2 (5)	0 (0)	
ESSDAI score, mean (range)	3 (0–10)		
Active disease (ESSDAI ≥ 4)	9		
Serological features, *N* (%)			
ANA	31 (79)	0 (0)	
Anti-SSA/Ro	33 (85)	0 (0)	
Anti-SSB/La	21 (54)	0 (0)	
Anti-SSA/Ro and anti-SSB/La	20 (51)	0 (0)	
Low serum C3	19 (49)	0 (0)	
High serum IgG	16 (41)	0 (0)	
CRP	22 (56)	0 (0)	
ESR	27 (69)	0 (0)	

## Data Availability

The data used and/or analyzed in the current study are available from the corresponding author on reasonable request.
